# A dual-polarized RIS-assisted spatial modulation architecture for robust vehicular communications in urban environments

**DOI:** 10.1371/journal.pone.0356140

**Published:** 2026-08-14

**Authors:** Shane E. Lewis, M.P. Darshan, Praveen Kumar, Goutham Simha G.D.

**Affiliations:** 1 Manipal Institute of Technology, Manipal Academy of Higher Education, Manipal, India; 2 Department of Electronics and Communication Engineering SDM Institute of Technology (SDMIT) Ujire, Karnataka, India; Beijing Technology and Business University, CHINA

## Abstract

With Vehicle-to-Vehicle (V2V) communication being one of the enabling elements for intelligent transportation systems and autonomous driving, it enables a reliable one-to-one exchange of safety-related information, including vehicle speed, inter-vehicular distance, braking systems, and the surrounding roadway environment. The highly dynamic nature of vehicular environments, combined with stringent reliability, latency, and energy-consumption requirements, makes it natural to adopt communication architectures that are both spectrally and hardware- and power-efficient. In this context, the current paper explores an intelligent surface (RIS) aid V2V communication system that makes use of a suggested dual-polarized spatial modulation (DPSM) scheme employing one active RF chain per symbol interval over four physical antennas. The suggested architecture makes use of spatial, polarization, and RIS-generated degrees of freedom to augment the adaptability of linkages with significantly lower RF hardware complexity and power usage. Detailed system-level simulations are conducted in realistic conditions, utilizing 3GPP V2V/V2X fading channel models that incorporate vehicle mobility and urban propagation conditions. Major performance indicators, such as received signal strength indicator (RSSI), reference signal received power (RSRP), reference signal received quality (RSRQ), bit error rate (BER), and signal-to-noise ratio (SNR), as well as energy efficiency, are strictly measured and compared with conventional full-MIMO performance. Simulation results demonstrate that the RIS-assisted single-active-RF-chain DPSM scheme can achieve significant energy efficiency gains while maintaining competitive BER performance across a wide SNR range. Where, the transmitter uses physical dual-polarized antennas which provide 8 logical spatial-modulation ports, i.e.,4 antennas× 2 polarizations, but only one RF chain and one logical port are active in each symbol interval. These enhancements in RSSI, RSRP and RSRQ highlight the ability of RIS to provide signal shaping and polarization diversity to reduce the harsh fading and blockage characteristics of the V2V operating environment in the urban environment. Although the full-MIMO system exhibits better BER performance due to its increased spatial diversity, the proposed DPSM architecture offers a more advantageous trade-off between performance, complexity, and energy. These results support the use of the RIS-supported DPSM architecture as a highly suitable and convenient design for next-generation V2V communications, particularly on systems with power- and cost-limited vehicle platforms.

## 1. Introduction

Reconfigurable intelligent surfaces have been a disruptive enabling technology for future wireless systems, enabling low-cost passive control of the propagation environment through element-wise phase (and amplitude) adjustments. RIS can significantly improve coverage, spectral efficiency, and energy efficiency by shaping wavefronts and creating favourable cascaded channels between transmitter and receiver [1–3]. The majority of early RIS research was conducted under the assumption that single-polarization elements were used and only simple phase control was applied. Recent advances in this area exploit polarization diversity at the surface itself, i.e., dual-polarized RIS to add an orthogonal degree of freedom that increases design flexibility, coverage uniformity, and resilience to polarization mismatch in practical deployments [4,5].

Two challenges are particularly important for realizing the full potential of dual-polarized RIS in dynamic scenarios such as vehicular networks. First, practical V2V links experience severe small-scale fading, non-Gaussian scattering, and non-stationarity (notably Doppler due to mobility). Nakagami-m distributions often provide an accurate fit for V2V small-scale fading across a range of environments and are widely used in the analysis and simulation of vehicular links [6–8]. Second, realistic large-scale propagation should be anchored to physical reference distances—the close-in (CI) free-space reference model is widely adopted in mmWave and vehicular studies because it preserves physical scaling and enables meaningful cross-scenario comparison [8,9].

Another practical impairment observed in many V2V and constrained-aperture environments is the keyhole (or pinhole) effect, in which rich multipath at each end does not translate into full MIMO rank at the link level, producing effectively low-rank channels even when spatial correlation is low. This phenomenon greatly limits multiplexing gains unless countermeasures such as environment reshaping via RIS are used [10,11]. The Doppler spread and channel time variations due to mobility also make coherent combining across the RIS elements challenging and channel estimation and phase-control strategies complex [12,13]. A promising solution to improve robustness in polarization and time-varying environments is dual-polarized RIS hardware combined with specially designed configuration sequences having excellent autocorrelation/cross-correlation properties. Golay complementary sequence pairs (and their multi-dimensional extensions) are classical constructs that guarantee perfect aperiodic autocorrelation cancellation and have been employed in communications and radar for low-PEP signaling, low-PMEPR signaling, and channel-training designs.

Recently, Golay-pair-based configurations have been suggested for RIS phase indexing across orthogonal polarizations to create broad flat or Doppler robust beam patterns and to provide enhanced channel estimation reliability for cascaded channels [14]. Bringing these strands together (i) dual polarized RIS hardware, (ii) Golay pair-based configuration, (iii) realistic V2V channel statistics (Nakagami small-scale fading, CI large-scale loss), (iv) keyhole-type low-rank effects, and (v) Doppler time variation yields a system design space that is rich and practically relevant for future vehicular platforms. The combined system can exploit polarization degrees of freedom to recover diversity lost to keyhole constraints; (b) use Golay pair patterns in reducing training overhead while maintaining low cross-interference between polarizations; and (c) adopt CI path loss and Nakagami-m models over the Doppler frequency create realistic performance predictions in V2V scenarios. Realization of these gains necessitates careful joint design of RIS element mapping, polarization routing, Golay-based training sequences, and time-synchronous phase updates that account for Doppler and imperfect CSI; evaluation should use both analytical models as well as measurement-based or high-fidelity simulation of vehicular channels. Existing RIS assisted vehicular communication investigations have largely been focused on conventional beamforming and/or standard MIMO architecture with multiple RF chains.

The following paper aims to address this gap by creating a viable vehicle-to-vehicle (V2V) communication link in an urban setting, exploiting the accuracy of reconfigurable intelligent surfaces (RIS) without compromising the energy efficiency of a dual-polarised spatial modulation multiple-input multiple-output (MIMO) system [15,16]. Simple real-time tuneable RIS operation can be used in urban V2V conditions to reduce or effectively eliminate the effects of multipath fading caused by relative motion between the receiver and transmitter, with time dependent co-phasing or selectively phased reflected signals used to cause a desirable combination of arrivals or cancellation of harmful components between multiple paths effectively reducing the effects of the Doppler shifts as well by compensating the Doppler delay of individual propagation paths [14–16]. Moreover, RIS orthogonal polarized elements (which allow the synthesising of a wide beam) can be engineered with phase-shift vectors of the two polarizations by constructing an orthogonal pair of RIS phase-shift structure vectors; so, having RIS phase-shift vectors as Golay pair will enable the projection of a large beam that can service a large number of users simultaneously and also can increase the size of the RIS coverage region [17,18]. The architecture can be combined with a sub-optimal greedy algorithm based on maximum-energy, and maximum-likelihood detectors can be used in the RIS based spatial modulation framework, providing the possibility of high spectral efficiency even at low SNR levels, but with less provisional spectral efficiency at high SNR. In [19] the propagation characteristics and path loss modelling on a large scale were examined and the accuracy, sensitivity and stability of the parameters were investigated under various propagation conditions. Comprehensive specifications for NR-based V2X services are given in the 3GPP standard TR 38.886 [20] which defines requirements for user equipment transmissions and receptions for reliable vehicular communication scenarios. There has been a recent surge in a new generation of communication techniques that can be implemented with RIS technology, capable of increasing spectral efficiency and signal reliability in next-generation wireless systems. To achieve this, Liu et al. [21] presented an RIS-assisted dual-polarized spatial modulation framework with CVCNN detector, achieving better detection performance and transmission efficiency.

Recent studies have explored the application of RIS technology in urban 5G environments, with a particular focus on enhancing spectral efficiency through hybrid beamforming and intelligent precoding. The authors in [22] implemented RIS assisted hybrid beamforming for V2I communication in urban 5G VR networks proving the potential of RIS in improving link reliability over dense propagating channels

*A. Our Contributions:*

1. The present paper proposes a novel RIS structure based on a dual-polarised Golay pair-enhanced RIS integrated with single-chain spatial modulation to improve energy efficiency.
2. We propose a novel RIS phase-steering technique, whereby, to optimize the coherent combination at the receiver and remove out-of-beam sidelobes, the Golay pairs are combined with offsets and phase-encoded via the SVD.
3. An optimization scheme based on greedy detection is developed, where the selection of both the RIS and antenna ports is determined by the optimal RIS position and the spatial modulation port that provides the maximum energy gain.
4. We perform a detailed simulation analysis of the proposed RIS-enhanced dual-polarised spatial modulation model and compare it with 4 × 4 MIMO baseline schemes, considering a realistic vehicle-to-vehicle channel with Nakagami fading, keyhole and rain spread effects, as well as Doppler spread.

**Fig 1:**
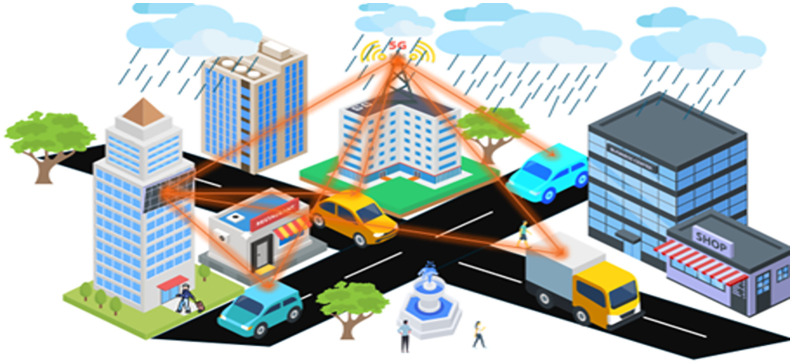
RIS-Assisted Polarization-Aware V2V Communication in a Rainy Smart City Environment.

**Fig 2:**
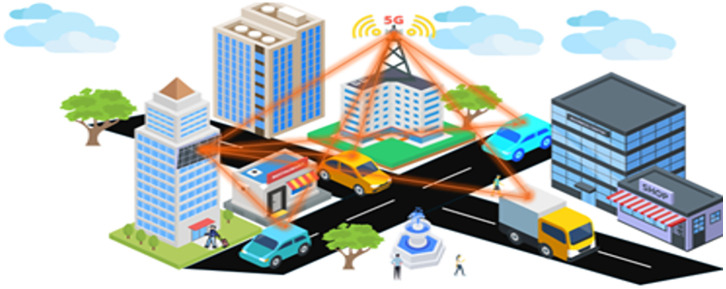
RIS-Assisted Polarization-Aware V2V Communication in a typical 5G Smart City Environment.

Fig 1 and Fig 2 provide a high-level overview of the V2V Urban scenario modeled and simulated in this paper, with rain effects included and standard environment effects considered. An urban city grid is established, along with predefined vehicle trajectories and speeds. This simulation takes into account all four faces of each building as potential candidates for placing the RIS panel, using a greedy algorithm to determine the optimal position for RIS given the vehicle position as an optimization problem. Both LOS and NLOS paths are probed and combined at the receiver, with realistic urban conditions accounted for in this simulation.

## 2. System and channel models

### *A. Spatial Modulation:* [1,4,7]

Spatial modulation is a technique that activates only one transmit antenna per symbol, mapping the information into both a conventional constellation and the index of the active antenna. This modulation technique produces higher spectral and energy efficiency while reducing the RF chain count. Drawing from this spatial modulation can be extended to accommodate co-located orthogonally polarized elements at each location. Each transmit antenna effectively has two polarization ports, that is, vertical and horizontal, thus enhancing the spatial modulation architecture without affecting the physical footprint. Based on the above model, the vehicles transmit using a single active RF chain per symbol while selecting one of the 8 logical ports formed by 4 physical dual-polarized antenna locations. Thus, the hardware contains 4 physical antenna locations, but only one RF chain is active at a time. In the following simulations, we evaluate BER, SNR, energy efficiency, RSSI, and RSRP for the two mobility scenarios, namely towards and away. Gaining insights from the above model, simulations in this study considers the vehicles to transmit using a single RF chain while simultaneously switching between dual-polarized ports. We implement an ML detection algorithm at the receiver aided by RIS reflections and analyze BER vs. SNR, Energy Efficiency, RSSI, and RSRP performance for two mobility-based scenarios, i.e., towards and away.

### *B. Dual-Polarized RIS Design* [15]

Recent advances in RIS architectures have introduced dual-polarized RIS panels, where each metaatom is capable of independently reflecting horizontal (H) and vertical (V) polarized signals. In our V2V system, we leverage this structure to exploit polarization diversity and improve beam coverage in environments with angularly dispersed users or vehicles. Each RIS element now supports two phase control channels:

$\phi_{H}\in C^{M}$: phase shifts applied to horizontally polarized waves

$\phi_{V}\in C^{M}$: phase shifts applied to vertically polarized waves

The signal received from a dual-polarized reception user becomes:


$$\begin{array}{c} r_{i}=\sqrt{P}{𝐠}_{i}^{T}\Phi_{i}{𝐡}_{i}x+n_{i},\;i\in \{H,V\} \end{array}$$ (1)

where:

${𝐡}_{i},{𝐠}_{i}\in C^{M}$: RIS-to-transmitter and RIS-to-receiver channels for polarizationi

$\Phi_{i}=\text{diag}\left(e^{j\phi_{i,\text{1}}},\ldots ,e^{j\phi_{i,M}}\right)$: phase configuration matrix

x: transmitted data symbol (BPSK is used here for the scalar RIS phase model, the full SM system uses 32-QAM as described in Section 3), $n_{i}\sim \text{CN}\left(\text{0},\sigma^{\text{2}}\right)$: complex AWGN

We apply Maximal Ratio Combining (MRC) across both polarizations at the receiver. The total SNR becomes:


$$\begin{array}{c} \text{SNR}=\frac{P\beta_{h}\beta_{g}}{\sigma^{\text{2}}}G_{\text{0}}(\tilde{\phi })G_{\text{0}}(\phi )\text{A}(\phi ) \end{array}$$ (2)

where the power-domain array factor (PDAF) is:


$$\begin{array}{c} A(\phi )={\left|\phi_{H}^{T}\left({𝐚}_{H}(\tilde{\phi })\circ {𝐚}_{H}(\phi )\right)\right|}^{\text{2}}+{\left|\phi_{V}^{T}\left({𝐚}_{V}(\tilde{\phi })\circ {𝐚}_{V}(\phi )\right)\right|}^{\text{2}} \end{array}$$ (3)

To produce a broad beam and achieve polarization diversity, we co-design the RIS phase shifts $\phi_{H}$ and $\phi_{V}$ to satisfy the broad-beam condition:


$$\begin{array}{c} R_{\phi_{H}}[\tau ]+R_{\phi V}[\tau ]=\text{2}M\delta [\tau ] \end{array}$$ (4)

where $R_{\phi }[\tau ]$ is the aperiodic autocorrelation function of ϕ, and δ[τ] is the Kronecker delta.

This condition is met by constructing $\phi_{H}$ and $\phi_{V}$ as a Golay complementary pair, i.e., sequences whose autocorrelations cancel out at all lags except zero


$$\begin{array}{c} R_{n}[\tau ]+R_{v}[\tau ]=\text{2}M\delta [\tau ] \end{array}$$ (5)

Such Golay pairs flatten the beam pattern over all angles, enabling wide angular coverage. This design is particularly effective in mobile V2V setups where users (vehicles) may move through wide angular areas.

In our simulations, we represent dual-polarized RIS by splitting the RIS elements into H and V polarization groups. Beamforming vectors are generated independently for each polarization, and MRC is performed at the receiver to combine their contributions. This dual-polarized RIS model enables better link reliability and angular robustness in high-mobility vehicular networks.

A planar RIS with N elements are deployed at the midpoint of the track. Each element supports horizontal (H) and vertical (V) polarizations.

Let


$$\begin{array}{c} {𝐇}_{l}^{\left(p\right)}(t)\in C^{N\times M_{l}},\;{𝐇}_{r}^{\left(p\right)}(t)\in C^{M_{l}\times N},\;p\in \{\text{H},\;\text{V}\} \end{array}$$ (6)

denote the Tx → RIS and RIS → Rx fading matrices for polarization p. We compute per-element phase shifts by


$$\begin{array}{c} \theta_{n}=∠(\underset{\mu_{n}^{\left(II\right)}}{\underbrace{{𝐡}_{r,n}^{(H)H}{𝐡}_{i,n}^{\left(H\right)}}}+\underset{\mu_{n}^{\left(V\right)}}{\underbrace{{𝐡}_{r,n}^{(V)H}{𝐡}_{i,n}^{\left(V\right)}}}) \end{array}$$ (7)

where ${𝐡}_{t,n}^{\left(p\right)}$ is the nth row of ${𝐇}_{t}^{\left(p\right)}$ and ${𝐡}_{r,n}^{\left(p\right)}$ the nth column of ${𝐇}_{r}^{\left(p\right)}$. The RIS phase matrix is


$$\begin{array}{c} \Theta =\text{diag}\left(e^{-j\theta_{\text{1}}},\ldots ,e^{-j\theta_{N}}\right) \end{array}$$ (8)

The dual-polarized RIS reflection combines both polarizations:


$$\begin{array}{c} {𝐇}_{\text{RIS}}(t)=\sum_{p\in \{\text{H},\;\text{V}\}}\;{𝐇}_{r}^{\left(p\right)}(t)\Theta {𝐇}_{i}^{\left(p\right)}(t). \end{array}$$ (9)

### 2.1 Dual polarized spatial modulation: [15,19–21]

Modern Vehicle-to-Vehicle (V2V) communication systems often struggle with limited energy resources and fairly complex hardware. New technologies such as reconfigurable intelligent surfaces (RIS) and spatial modulation (SM) help address these issues. RIS enables passive beamforming by adjusting how incoming signals reflect off its programmable elements. SM, on the other hand, reduces the number of RF chains needed by embedding information in the index of the active transmit antenna. When an RIS uses dual-polarized elements represented by a 2 × 2 Jones matrix, it adds even more flexibility by allowing independent control of two orthogonal polarizations. This section outlines the system models and compares how dual-polarized SM paired with RIS performs against a 4-stream full MIMO baseline that uses QPSK modulation and MMSE detection. QPSK is adopted as the baseline modulation scheme used since it provides a favorable tradeoff between robustness and spectral efficiency due to its simple constellation structure which is effective for isolating the effects of channel fading, RIS phase alignment and receiver processing

The baseband received vector is


$$\begin{array}{c} y=\left(H_{𝐝𝐢𝐫\;}+H_{𝐫𝐢𝐬\;}\right)x+n, \end{array}$$ (10)

where $H_{d\text{ir}}\in C^{n_{r}\times n_{u}},x\in C^{n_{\varepsilon }}$, and $n\sim CN\left(\text{0},\sigma_{w}^{\text{2}}I\right)$.

For element k, define

- $H_{\text{1},k}\in C^{\text{2}\times n_{k}}$: channel from transmitter ports to the element’s V/H ports.
- $H_{\text{2},k}\in C^{n,\times \text{2}}$: channel from the element’s V/H ports to receiver ports.
- $R_{k}\in C^{\text{2}\times \text{2}}$: Jones matrix of element k.

Element contribution:


$$\begin{array}{c} H_{\text{elem },k}=H_{\text{2},k}R_{k}H_{\text{1},k}\in C^{\text{2}k_{r}\times n_{k}} \end{array}$$ (11)

Total RIS channel can be written as:


$$\begin{array}{c} H_{\text{ris }}=\sum_{k=\text{1}}^{K}\;H_{\text{elem },k} \end{array}$$ (12)

A practical Jones parametrization with small cross-polar leakage ε is [14–16]


$$\begin{array}{c} R_{k}=[\begin{array}{cc} e^{j\phi_{V,k}} & \varepsilon e^{j\left(\phi_{V,k}+\delta \right)}\\ \varepsilon e^{j\left(\phi_{H,k}+\delta \right)} & e^{j\phi_{H,k}} \end{array}] \end{array}$$ (13)

with $\phi_{V,k},\phi_{H,k}$ tunable and δ a small implementation-dependent offset. In the scalar RIS (single polarization) case, we can write $R_{k}=e^{j\phi \text{k}}$.

Construct $M\in C^{\left(n_{r}n_{k}\right)\times K}$ with column $k=\text{vec}\left(H_{\text{2},k}H_{\text{1},k}\right)$. The SVD


$$\begin{array}{c} M=U\Sigma V^{H} \end{array}$$ (14)

yields right singular vectors V. The principal vector V(:,1) suggests a base-phase per element:


$$\begin{array}{c} \phi_{\text{base};\;k}=∠\left(V_{k}(\text{1})\right)+\phi_{\text{Doppler }}. \end{array}$$ (15)

Per-polarization total phases may combine base-phase with phase codes ($\phi_{V,k}^{\text{code }},\phi_{H,k}^{\text{code }}$):


$$\begin{array}{c} \phi_{V,k}=\phi_{\text{base },k}+\phi_{V,k}^{\text{code }},\;\phi_{H,k}=\phi_{\text{base },k}+\phi_{H,k}^{\text{code }}. \end{array}$$ (16)

Spatial modulation transmitter SM activates a single logical port per symbol interval. For antenna index $a\in \left\{\text{1},\ldots ,n_{i}\right\}$ mapped from spatial bits and symbol s∈S,


$$\begin{array}{c} x={𝐞}_{a}s, \end{array}$$ (17)

with spectral efficiency $R_{\text{SM}}={\text{log}}_{\text{2}}\left(n_{t}\right)+{\text{log}}_{\text{2}}|S|$.

A full MIMO transmitter may simultaneously send $n_{L}$ symbols. The MMSE linear receiver uses


$$\begin{array}{c} W={\left(H_{\text{tot}}^{H}H_{\text{tot}}+\frac{\text{1}}{\text{SNR}}I_{n_{\text{t}}}\right)}^{-\text{1}}H_{\text{tot}}^{H},\;H_{\text{tot}}=H_{\text{dir}}+H_{\text{RIS}} \end{array}$$ (18)

Estimated symbol vector $\hat{x}=Wy$.

Joint ML detection over antenna index and symbol:


$$\begin{array}{c} (\hat{a},\hat{s})=\text{arg}\underset{a\in \left\{\text{1},\ldots ,m_{l}\right\},x\in S}{\text{min}}\;{\left\|y-\left(H_{\text{dir }}+H_{\text{RIS }}\right)\left({𝐞}_{a}s\right)\right\|}^{\text{2}}. \end{array}$$ (19)

Integrating SM and Dual Polarization allows the system to have the following benefits:

Logical port augmentation with limited RF chains: Dual polarization yields two logical ports per physical antenna (V/H). SM encodes index bits using a single active RF chain, enabling ${\text{log}}_{\text{2}}\left(n_{t}\right)$.

### 2.2 Integration of RIS into V2V Infrastructure: [19,20]

In the V2V simulation modelled for the analysis, we assume a 64-element RIS. This is chosen as it captures the main benefits of surface-assisted beamforming without additional complexity. This size offers sufficient degrees of freedom for reflection, which enhances signal concentration and hence improves link reliability for V2V communications while simultaneously being computationally tractable.

Let L=64, denote the number of RIS elements. The small-scale fading matrix from the $N_{t}$ transmit antennas to the RIS is modeled as


$$\begin{array}{c} {𝐇}_{T\to 𝐑𝐈𝐒}\in {𝐂}^{L\times N_{t}} \end{array}$$ (20)

and similarly, the channel from the RIS to the $N_{r}$ receive antennas is


$$\begin{array}{c} {𝐇}_{𝐑𝐈𝐒\to R}\in {𝐂}^{N_{r}\times L} \end{array}$$ (21)

both under Nakagami- m fading and path loss [7]

The RIS applies a diagonal phase-shift matrix.


$$\begin{array}{c} \Phi =𝐝𝐢𝐚𝐠\left(e^{j\theta_{1}},e^{j\theta_{2}},\ldots ,e^{j\theta_{L}}\right)\in {𝐂}^{L\times L} \end{array}$$ (22)

where $\theta_{\text{𝓁}}$ is the phase change induced by the 𝓁 th element.

Under far-field planar wave assumptions, the overall cascaded RIS-assisted MIMO channel is


$$\begin{array}{c} {𝐇}_{\text{RIS}}={𝐇}_{\text{RIS}\to R}\Phi {𝐇}_{T\to \text{RIS}}\in C^{N_{r}\times N_{t}} \end{array}$$ (23)

### 2.3. Keyhole and Nakagami Modeling: [7,10]

Field measurements indicated a growing need for statistical channel models in cities, towns, and rural areas to support the development of terrestrial mobile communication services, automated driving systems, and other applications. The Nakagami-m distribution is well-suited to describe fading conditions associated with various communication media, atmospheric propagation, out-of-sight microwave, terrestrial scatter, and interference enhancement from neighbouring or adjacent environments. Various measurement methodologies are used to analyse channel characteristics in terms of different scalar parameters.

Research indicates that vehicle-mounted communications, travelling along straight, unobstructed road sections lasting several minutes without modulation regime changes, exhibit Nakagami-m fading characteristics. Nakagami-m modelling is well-suited to urban and suburban environments where rooftop obstructions predominate, especially for a vehicle traversing open environments with obstructed paths on adjacent roads. Urban and suburban fast-fading scenarios, which occur in the absence of reflection or large shadowing, likewise exhibit the Nakagami-m model. High-speed, straight vehicular communications in suburban areas, spanning several hundred meters, remain compatible with the Nakagami-m formalism without modulation regime or power-class switches.

#### 2.3.1. Mathematical modeling.

Nakagami- m is a flexible parametric model for small-scale fading amplitudes. It can model a wide range of fading severities:

m=1→ Rayleigh fading (rich scattering, zero mean complex Gaussian).

m>1→ less severe fading (peakier amplitude distribution; approximates Rician-like behavior). 0.5≤m<1→ more severe than Rayleigh.

Two parameters define the distribution: the shape m>0.5 and the spread (scale) ω>0. In practice m captures fading severity and is often estimated from measurements.

If R is the envelope (amplitude) of a Nakagami channel, its PDF is [7,10]


$$\begin{array}{c} f_{R}(r)=\frac{\text{2}m^{m}}{\Gamma (m)\omega^{m}}r^{\text{2}m-\text{1}}\text{exp}\left(-\frac{m}{\omega }r^{\text{2}}\right),\;r\geq \text{0} \end{array}$$ (24)

where Γ(·) is the Gamma function. The corresponding instantaneous power $P=R^{\text{2}}$ follows a Gamma distribution:


$$\begin{array}{c} f_{P}(p)=\frac{m^{m}}{\Gamma (m)\omega^{m}}p^{m-\text{1}}\text{exp}\left(-\frac{m}{\omega }p\right),\;p\geq \text{0} \end{array}$$ (25)

Thus E[P]=ω and $\text{Var}(P)=\frac{\omega^{\text{2}}}{m}$.

These relations are commonly used to set ω (power scaling) and to interpret m (fading spread). Given measured envelope samples $\left\{r_{i}\right\}$, one may estimate m and ω by moment matching:


$$\begin{array}{c} \hat{\omega }=\frac{\text{1}}{N}\sum_{i}\;r_{i}^{\text{2}},\;\hat{m}=\frac{{\hat{\omega }}^{\text{2}}}{\frac{\text{1}}{N}\sum_{i}\;\;{\left(r_{i}^{\text{2}}-\hat{\omega }\right)}^{\text{2}}} \end{array}$$ (26)

Maximum-likelihood estimation is utilized for precision.

**Algorithm 1** assesses the RSSI and RSRP performance of both RIS-assisted and direct V2V links as a function of the V2V transmitter–receiver distance and the sampling frequency. For a given distance, the path loss is determined using a distance and frequency-dependent propagation model. The Doppler shifts for relative vehicle mobility in the same and opposite directions are calculated from the relative vehicle velocity and the wavelength. For the RIS-assisted scenario, the effective cascaded RIS channels are generated, and the received power is calculated by the product of channel gain, transmit power, and path-loss. RSSI is derived from the received power, whereas RSRP is evaluated by normalizing RSSI with respect to the number of resource blocks. This procedure is then repeated for the direct V2V link, considering Nakagami fading, Doppler spread, and keyhole effects. Finally, the RSSI and RSRP for each considered frequency sample are averaged to obtain the performance metrics for each distance. This algorithm allows for a realistic comparison of RIS-assisted and non-RIS V2V links considering realistic fading, Doppler and propagation loss scenarios.

**Algorithm 1: Compute RSSI and RSRP for RIS-Assisted and Direct V2V Links**

**Require:** ***Nt, Nr, Lris, mNLOS, Nsub, freq[], dist[], v_bs, v_me, Pt, c, numRBs***

**Ensure:** ***RSSI_ris_same[], RSSI_ris_opp[], RSRP_ris_same[], RSRP_ris_opp[],***

***RSSI_no_same[], RSSI_no_opp[], RSRP_no_same[], RSRP_no_opp[]***

1: ***for each d in dist do***

2:   pathLoss_dB(f) ← 20 log10(d) + 20 log10(f) − 147.55

3:   ***for each f in freq do***

4:   λ ← c/ f

5:   fD_tow ← |v_me + v_bs|/ λ

6:   fD_awa ← |v_me − v_bs|/ λ

7:   t_snap ← randUniform(0, 1)

8:   (H_tow, H_awa) ← generateRISChannel

9:   Pr_tow ← ||H_tow||²· Pt·${\text{1}\text{0}}^{-\frac{pathLoss_{dB}}{\text{1}\text{0}}}$

10:   Pr_awa ← ||H_awa||²· Pt·${\text{1}\text{0}}^{-\frac{pathLoss_{dB}}{\text{1}\text{0}}}$

11:   RSSI_tow ← 10 log10(Pr_tow)

12:   RSSI_awa ← 10 log10(Pr_awa)

13:   RSRP_tow ← RSSI_tow − 10 log10(numRBs)

14:   RSRP_awa ← RSSI_awa − 10 log10(numRBs)

15: end for

16:   RSSI_ris_same ← mean(RSSI_tow)

17:   RSSI_ris_opp ← mean(RSSI_awa)

18:   RSRP_ris_same ← mean(RSRP_tow)

19:   RSRP_ris_opp ← mean(RSRP_awa)

20: end for

21:   ***for each d in dist do ◊ Direct path***

22:     repeat pathLoss

23:   ***for each f in freq do***

24:     H_same ← Nakagami + Doppler(fD_tow)

25:     H_opp ← Keyhole + Doppler(fD_awa)

26:  compute Pr_tow, Pr_awa, then RSSI, RSRP as above

27: end for

28:   RSSI_no_same ← mean(RSSI_tow)

29:   RSSI_no_opp ← mean(RSSI_awa)

30:   RSRP_no_same ← mean(RSRP_tow)

31:   RSRP_no_opp ← mean(RSRP_awa)

32: end for

## 3. Integration of Spatial Modulation to RIS Assisted V2V model [19,20]

We consider the same RIS-assisted V2V model as discussed in the previous section, with the inter-vehicular distance, d∈[10,1000] m with RIS placement fraction, α∈[0.1,0.9] along the line of sight. At any given instant of time, t, exactly one of the 8 logical transmit ports formed by 4 physical antenna locations and 2 polarisations per location is activated based on the input bits. This results in only 1 RF chain active per symbol interval. Hence, the transmit vector is


$$\begin{array}{c} x=e_{p}s,\;p\in \{\text{1},\text{2},\text{3},\text{4}\}, \end{array}$$ (27)

where $e_{p}$ is the p th column of the 4×4 identity matrix. This single-RF large-scale SM principle both relaxes the RF chain count and exploits spatial constellation for extra bits.

In order to ensure a nominal per-link SNR of $\Gamma_{\text{tur }}=\text{10}\;\text{dB}$, the effective power is limited to


$$\begin{array}{c} P_{\text{aII}}=\text{min}\left\{P_{l},P_{l}\frac{\Gamma_{\text{Lar}}}{P_{r}/\sigma^{\text{2}}}\right\} \end{array}$$ (28)

Drawing from this, we then compute the energy efficiency of this simulation model using the following equation

$\text{EE}=\frac{R}{P_{\text{L}\text{t}\text{otal }}},$ where R, received bits, is defined as$R={\text{log}}_{\text{2}}\left(\text{1}+\Gamma_{\text{Lar}}\right)$

and $P_{\text{Ltotal }}$ is defined as


$$\begin{array}{c} \begin{array}{c} P_{\text{L}\text{t}\text{otal }}=P_{\text{ell }}+P_{\text{circ }}+\left(\text{64}\times P_{\text{RIS }}\right),\\ P_{\text{circ }}=\text{ per RF chain circuitry power },\;P_{\text{RIS }}=\text{ static per element RIS power}.\; \end{array} \end{array}$$

The percentage of EE gain is computed as:


$$\begin{array}{c} \text{ Gain }=\frac{{\text{EE}}_{\text{BM}}-{\text{EE}}_{\text{3}\;\text{L}}}{{\text{EE}}_{\text{3}\;\text{L}}}\times \text{100}\% \end{array}$$ (29)

In our simulation, transmitter RF impairments are captured via an Error Vector Magnitude (EVM) model, where the EVM parameter ε∈[0,1] specifies the ratio of the root mean square (RMS) error vector amplitude to the RMS reference signal amplitude. This simulation sets ε=0.05, effectively introducing 5% transmitter degradation to emulate a realistic scenario.

This simulation also explores the effect of RIS positioning on optimizing the SM-RIS assisted V2V infrastructure and proposes a joint optimization model framed over distance, RIS placement, and the optimal SM port.

For each vehicular separation d defined by the set


$$\begin{array}{c} d\in \{\text{10},\text{30},\ldots ,\text{1000}\}\text{m} \end{array}$$

a discrete search is performed over the RIS placement fraction α defined by the set


$$\begin{array}{c} \alpha \in \{\text{0}.\text{1},\text{0}.\text{15},\ldots ,\text{0}.\text{9}\} \end{array}$$

which locates the RIS at the distances $d_{\text{1}}=\alpha d$ from the transmitter vehicle and $d_{\text{2}}=(\text{1}-\alpha )d$ from the receiver vehicle. A similar search is performed over all possible SM ports p, and at each candidate (d,α,p) position, the energy efficiency is calculated, following which the most optimal tuple offering maximum energy efficiency is chosen.

In this environment, we compare a Baseline RIS-assisted V2V model with an SM-RIS-assisted V2V model, examining energy efficiency and antenna port gain for SM-RIS in each case.

**Algorithm 2** demonstrates the energy efficiency of the proposed RIS-assisted spatial-modulation system, accounting for optimal RIS placement. For each distance between the transmitter and receiver, the algorithm examines various RIS placement factors, α, where the segments αd and (1−α)d represent the distances from the transmitter to RIS and from RIS to receiver, respectively. For each considered RIS placement, path loss and Doppler shift are analyzed considering RIS-assisted aligned channel gains, along with CSI aging, finite phase quantization, and EVM impairment. The optimal spatial-modulation port is determined by the channel gain. Using the optimal port, the received power, achievable rate, and total power are calculated. The energy efficiency is defined as the ratio of achievable rate to total power. The RIS placement with the highest ratio is considered optimal for the given distance. The same process is applied to the same-direction V2V and opposite-direction V2V mobility cases, as well as to the baseline method. As a result, the algorithm enables a distance-specific assessment of the proposed SM–RIS system relative to the baseline system, focusing on energy efficiency.

Algorithm 2: Energy Efficiency Computation with Optimal RIS Placement

**Require:** ***Nt, Nr, Nsm, Lris, mNLOS, fc, c, distances[], alpha[], Pt_dBm, targetSNR_dB,***

***tauCSI, phaseBits, EVM, PcircSM, PcircBL, PrisElem, sigma2, Pint***

**Ensure:** ***EE_SM_same[], EE_SM_opp[], EE_BL_same[], EE_BL_opp[]***

1: $Pt\;\leftarrow \;{\text{1}\text{0}}^{\frac{Pt_{dBm}}{\text{1}\text{0}}}$, $\Gamma \;\leftarrow \;{\text{1}\text{0}}^{\frac{targetSNR_{dB}}{\text{1}\text{0}}},Q\;\leftarrow \;{\text{2}}^{phaseBits}$

2: ***for each d in distances do***

3:     λ ← c/ fc

4:   fD_same ← |v_me + v_bs|/ λ, fD_opp ← |v_me − v_bs|/ λ

5:     bestSMs, bestSMo, bestBLs, bestBLo ← −∞

6:     sumGainSI, sumGainOI ← 0

7:  ***for each α in alpha do***

8:       d1 ← α d, d2 ← (1 − α) d

9:       PL_dB ← 20 log10(d1) + 20 log10(fc) − 147.55

10:       $PL\;\leftarrow \;{\text{1}\text{0}}^{-\frac{PL_{dB}}{\text{1}\text{0}}}$

11:   // SM–RIS (same direction)

12:     gainsSI ← alignedChannelAvgAll(fD_same, tauCSI, Q, EVM)

13:     sumGainSI ← sumGainSI + gainsSI, p* ← arg max(gainsSI)

14:     Pr ← gainsSI[p*]· Pt· PL + Pint

15:     T ← min(Pt, Γ/ (Pr/ sigma2))

16:     R ← log2(1 + T)

17:     Ptot ← T + PcircSM + Lris· PrisElem

18:     bestSMs ← max(bestSMs, R/ Ptot)

19:   // SM–RIS (opposite direction) – similar using fD_opp

20:   // Baseline same (port = 1), Baseline opposite

21:  end for

22:  EE_SM_same[d] ← bestSMs, EE_SM_opp[d] ← bestSMo

23:  EE_BL_same[d] ← bestBLs, EE_BL_opp[d] ← bestBLo

24: end for

25: plot EE versus distance

**Fig 3:**
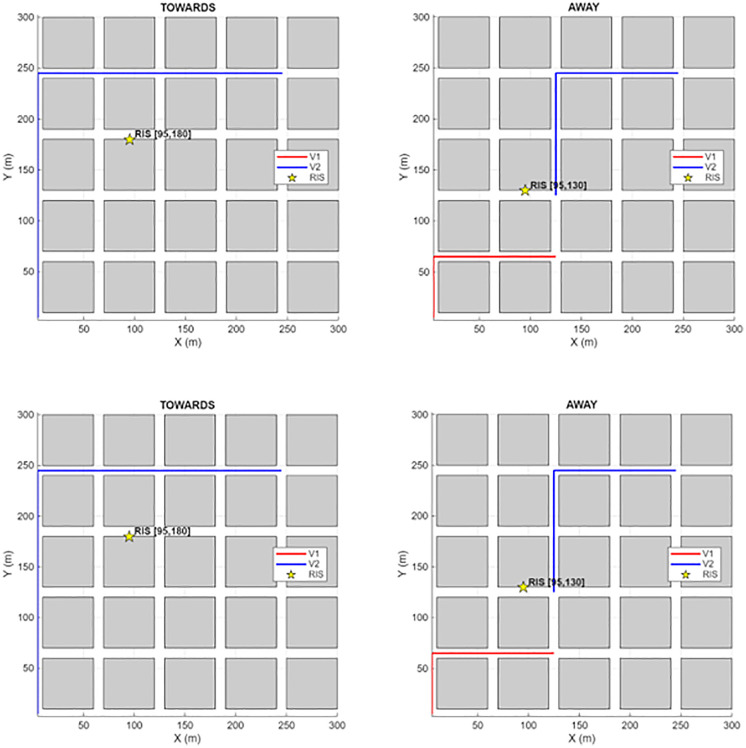
RIS placement in a typical V2V environment.

Fig 3 shows the two-panel city grid, which visualises the urban city layout and vehicle trajectories. The environment creates a 5x5 block grid, with a 50 m building separated by 10 m streets. Each building candidate contains 4 candidate RIS positions, i.e., midpoints of each side. A greedy algorithm is used to calculate the candidate position, which minimises the sum of the Euclidean distances to the time-averaged vehicle position. The trajectories of the vehicles are generated by finding the shortest paths between specified corner/edge nodes in a grid and then interpolating those node paths to form a smooth time series.

This algorithm computes the RIS site in the towards and away scenarios at coordinates [95, 180] and [95,130], respectively. These sites essentially balance the best RIS sites, balancing proximity to both vehicles under the greedy constraints in both simulation scenarios.

This study uses greedy optimization to determine the position of the RIS panel within the simulated city grid. A set of discrete RIS mounting coordinates, which are essentially the four faces per building, generated in the urban grid, which is defined by:


$$\begin{array}{c} C=\left\{c_{\text{1}},c_{\text{2}},c_{\text{3}},\ldots ..,c_{k}\right\}\subset {\mathbb{R}}^{\text{2}} \end{array}$$ (30)

Where, *C* is the finite set of RIS mounting coordinates

$c_{i}=\left(x_{i},y_{i}\right)\in R^{\text{2}}\;$, where each $c_{i}$ is the Cartesian coordinate, which signifies the horizontal placement of 1 RIS mounting location. These coordinates are represented in meters.$R^{\text{2}}$

is a two-dimensional Euclidean vector space. For each dynamic scenario, the time average positions of the two vehicles are computed as:


$$\begin{array}{c} m_{\text{1}}=\frac{\text{1}}{T}\;\int_{\;\text{0}}^{\;T}p_{\text{1}}(t)dt \end{array}$$ (31)

$$\begin{array}{c} m_{\text{2}}=\frac{\text{1}}{T}\;\int_{\;\text{0}}^{\;T}p_{\text{2}}(t)dt \end{array}$$ (32)

The RIS candidate c∈C that minimizes the Euclidean distance to these mean positions defined by


$$\begin{array}{c} =\text{arg}\;\underset{c\in C}{\text{min}}\left(\left|\left|m_{\text{1}}-c\right|\right|+\left|\left|m_{\text{2}}-c\right|\right|\right) \end{array}$$ (33)

This structure calculates the scalar cost function for each candidate RIS position and returns the candidate with the minimum cost. The selected discrete location is subsequently used as the fixed RIS coordinates for the given vehicle mobility scenario. This is a sub-optimal greedy algorithm designed for computational practicality.

The main rationale behind this setup in Fig 3 is to formulate an inexpensive, geometry-driven procedure that reduces the two-hop propagation distance, i.e., transmitter to RIS and RIS to receiver, over the given time window. The reduced propagation distance leads to lower CI path loss and larger per-hop amplitude factors, thereby increasing the average magnitude of the RIS reflected channel and, hence, the coherent gain achieved by phase alignment.

The greedy algorithm-based candidate selection is computationally efficient and trivial, which is immediately practical for robust vehicular motion since it optimises with respect to time-averaged positions. Producing a deterministic RIS positions a stable reference point for SVD-based phase extraction and Golay phasing used in the simulation pipeline.

## 4. Simulation results

The aim of the simulation is to understand how the RSSI, RSRP, RSRQ, and BER metrics vary with the SNR parameter in the proposed RIS-enabled DPSM model. RSSI indicates the total received power over a specified bandwidth in a communication channel, while RSRP considers only the power of the reference signals and is a key parameter in evaluating coverage. RSRQ is a dimensionless quantity that indicates the quality or long-term stability of the received signal based on both RSRP and RSSI. The BER reflects the error rates in the transmission of information over the communication channel.

The simulation takes place in a typical 5G V2V-based network. The environment is an urban microenvironment, with a shadowing standard deviation of 6 dB, and the channel is Nakagami-m, incorporating keyhole and CI models with Extended Pedestrian effects. The simulation setup comprises of ${\text{1}\text{0}}^{\text{6}}$ Monte Carlo iterations, and the main parameters are: an operating frequency of 3.5 GHz, with the considered 5G FR1 bandwidth of 100 MHz. We have compared the performance of a conventional 4 × 4 MIMO system without a RIS to that of a 4 × 4 MIMO system with a RIS in a typical V2V slow-fading scenario. Both perfect and imperfect CSI conditions are analyzed. In addition, the performance was compared with DPSM using Golay coding and SVD technique. It is expected that the metrics of interest will increase with higher SNR values and may show some degree of correlation with one another [1,19,20].

**Fig 4:**
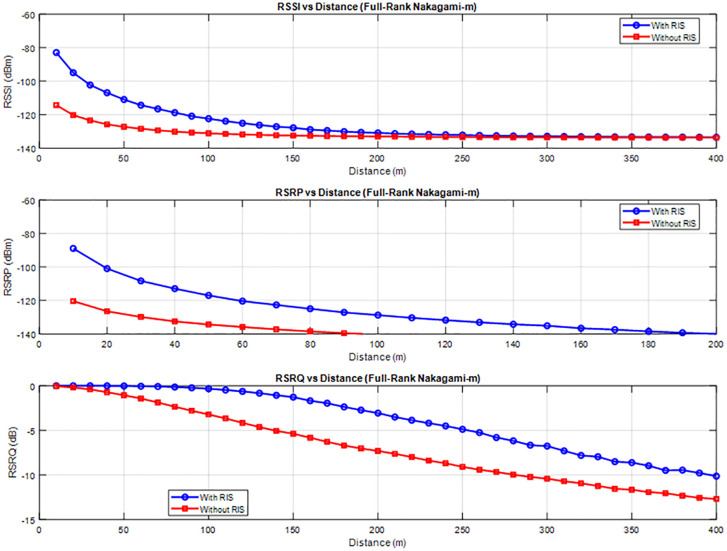
RSSI, RSRP, RSRQ vs Varying Distance over a Full Rank Nakagami Channel.

Fig 4 presents three plots that illustrate the changes in RSSI, RSRP, and RSRQ as a function of distance in a full-rank Nakagami-m fading channel, with and without a RIS. The results indicate that the received signal strength decreases monotonically with increasing distance between the transmitter and the receiver. The RIS-assisted connection has a significantly higher RSSI at all distances, especially in the near field (0,100m), where the RIS beamforming gain improvement is most crucial, and beamforming gain is most important.

The second plot illustrates the trend of RSRP versus distance. RIS assistance produces a better reference signal power, indicating a higher concentration of energy at the receiver. The degradation distance gradient is slightly steeper in the no-RIS case, indicating that RIS provides an effective path-strengthening effect that counteracts the negative effects of the Nakagami-m fading environment.

The third plot shows RSRQ versus distance, indicating that link quality is better; however, the RIS curve still shows a number of decibels higher than the no-RIS case, thus supporting the enduring link-quality benefit. Both cases deteriorate in RSRQ; however, the RIS curve clearly shows better performance, thus supporting the enduring link-quality benefit.

This emphasises the massive opportunity of the RIS-aided communication to ensure effective connectivity in a fading-prone wireless setting.

**Fig 5:**
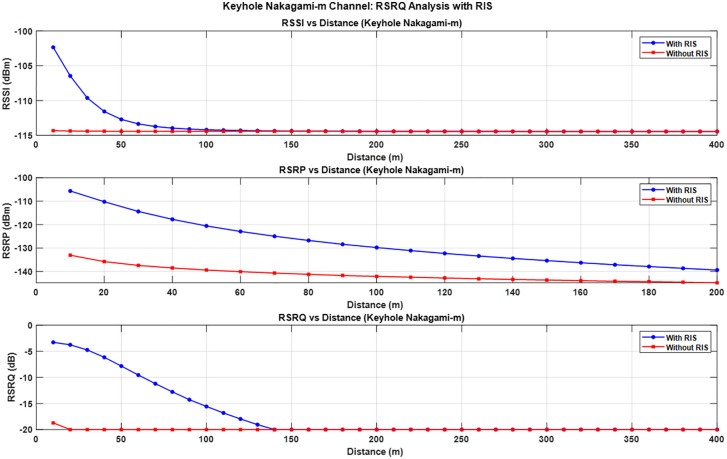
RSSI, RSRP, RSRQ vs Varying Distance over a Keyhole Nakagami Channel.

Fig 5 shows the RSSI, RSRP and RSRQ values over the keyhole Nakagami-m fading channel as a function of transmitter-receiver separation distance with and without RIS assistance.

To measure the impact of RIS deployment under keyhole propagation, we investigate all parameters over a distance range of 200–400 m. The keyhole model has two cascaded Nakagami-m fading processes, resulting in a rank-deficient MIMO channel with a limited spatial diversity and multiplexing gain for all antennas.

RSSI – Both setups fall off with distance and will attain the noise floor at ~80-100m. RIS assistance provides a small RSSI gain and slightly shifts the saturation point. The RIS assistance provides a small RSSI gain and pushes the saturation point away due to the additional scattering paths introduced by the RIS. This gain is not as large as it could be, since the limiting factor is the received channel rank, not the received power.

Consider RSRP: with RIS, the link decays approximately 35 dB over 200 m, whereas without RIS it decays suddenly at 80 m. The RIS optimizes the reflected signal phase to build an effective LoS-like path to compensate for some multipath combining that keyhole fading would cause. The RSRP gain of the RIS-assisted link is 25–30 dB over the range of 80–150 m, which is in line with the SNR gains in the BER curves.

For RSRQ: This is where the RIS gain is most apparent. The RIS-assisted link remains close to 0 to −5 dB up to ~140 m, while the non-RIS link is on the floor (~−19 dB) within the first 10–20 m. The RIS not only increases the received power but also improves the effective SINR, thereby reducing the impact of frequent destructive fading events on the keyhole channel.

**Fig 6:**
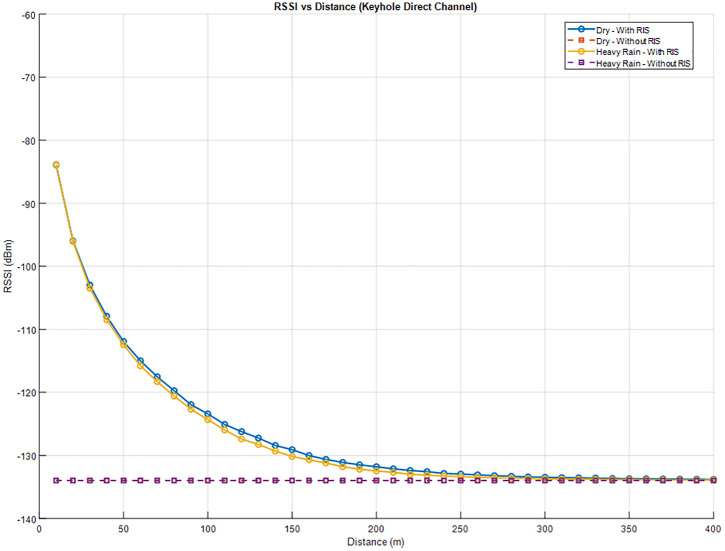
RSSI vs Varying Distance over a Keyhole Nakagami Channel with different weather conditions.

Fig 6 illustrates the behaviour of the RSSI in a keyhole direct channel environment, where spatial diversity is severely limited, making the received power highly sensitive to distance and channel blockage.

The RIS-aided curves in both dry and heavy-rain conditions have significantly higher RSSI values than the base non-RIS curves, which remain almost constant at around −133 dBm, indicating very weak, unrealistic signal intensities. These observations reveal that a direct keyhole connection is insufficient for maintaining high-quality communication, especially during rainy seasons.

The boost provided by RIS reflects its ability to alleviate the keyhole effect, thereby forming a new propagation route. Therefore, the figure indicates that RIS is necessary to restore link strength in a rank-deficient channel, thereby providing the channel with resilience against extreme weather conditions.

**Fig 7:**
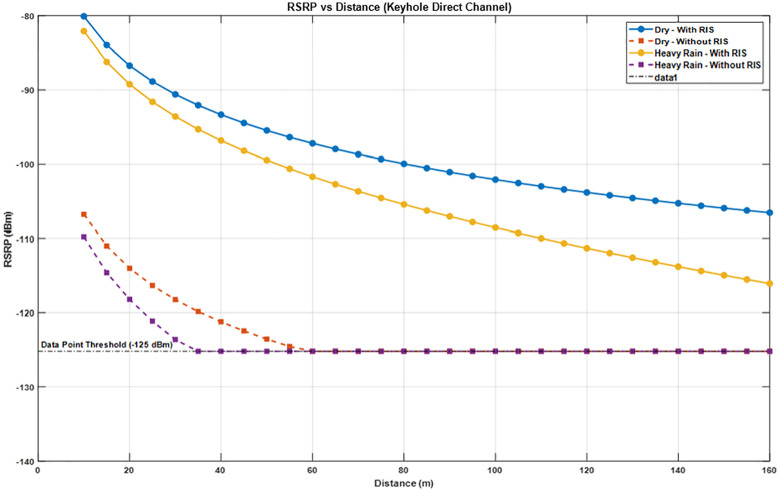
RSRP vs Varying Distance over a Keyhole Nakagami Channel with different weather conditions.

Fig 7 shows an extension of the RSRP analysis to a limited range of 10–160 m, with the addition of a −125 dBm data-point threshold, which is the minimum RSRP at which the reference signal can be successfully measured for subsequent synchronization and channel estimation. At 10 m, RSRP is at −80 dBm (dry) and −82 dBm (heavy rain), and with RIS assistance, the RSRP is slowly reduced to approximately −107 dBm at 160 m (dry) and −117 dBm at 160 m (heavy rain), which are well above the detection threshold throughout the entire simulated range. Beyond the −125 dBm threshold, both curves are steep due to the double-fading keyhole penalty, and the receiver no longer has a chance of discerning the reference signal: these are just measurement floor lines, not actual RSRP curves. This threshold crossing distance translates into a straight, measurable coverage measure: In dry weather, RIS assistance increases the reliably measurable range by about 2.9 × ; in heavy rain, by about 4.6 × , compared to the non-RIS keyhole baseline. The heavy-rain curves are consistently 2–10 dB lower than the dry curves for both RIS and non-RIS cases, which is consistent with the distance-proportional rain-specific attenuation overlaid on top of the keyhole path loss. Interestingly, the RIS/non-RIS difference increases with rain intensity (from ~34 dB at 10 m to over 20 m of coverage loss for the non-RIS link), suggesting that the keyhole channel and rain attenuation processes are both multiplicative and additive. The results illustrate the importance of RIS for achieving precise exploitation under degraded propagation conditions, where the coverage extension benefit of RIS deployment is multiplicative rather than an additive margin.

**Fig 8:**
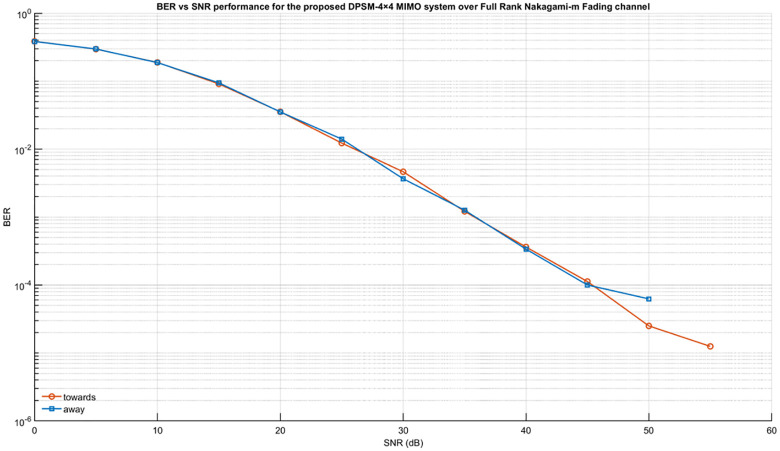
BER vs SNR performance for the proposed DPSM-4×4 MIMO system over Full Rank Nakagami-m Fading channel (with 3GPP path loss).

Fig 8 displays the BER vs. SNR for the proposed DPSM-4 × 4MIMO V2V links using QPSK, greedy RIS placement, SVD-based phase steering, and an MMSE receiver, with CI path loss, Doppler, and Nakagami fading. The curve shows monotonic decay, with a clear decline around 15dB, after which the BER drops rapidly, indicating coherent addition from the RIS-reflected field. The overlap between the BER curves of the two scenarios indicates that the greedy RIS placement yields a geometry-robust gain in both scenarios.

The steep decay after 15dB indicates that the beamforming gain and interference suppression by the RIS+MMSE model can be essentially interpreted, as the plot shows that RIS beam steering combined with MMSE equalization substantially reduces the SNR required to reach better BER targets.

**Fig 9:**
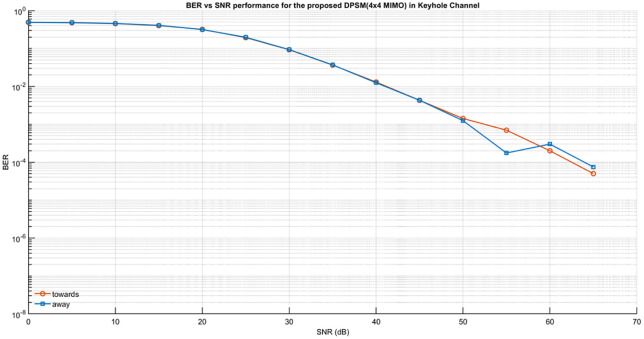
BER vs SNR performance for the proposed DPSM-4×4 MIMO system over Keyhole Nakagami-m Fading channel (with 3GPP path loss).

Fig 9 shows the BER vs. SNR for the implemented V2V model. The Golay complementary sequences introduce ±π offsets, whose complementary autocorrelation function cancels the accompanying sidelobes when the RIS reflections are combined across elements. Consequently, when combined with the SVD-derived phase base, the Golay offsets behave as a robustness layer, reducing out-of-beam sidelobe energy and mitigating sensitivity to small phase errors. Effectively, the SVD phase steers towards the dominant channel direction; the Golay pairs ensure the reflected power adds up coherently in the intended direction.

The BER curve also represents a visible decay in the mid SNR band at 25dB beyond which the error probability reduces rapidly. This decay represents the practical transmit antenna threshold above which the SVD-steered RIS, complemented by Golay pair offsets, begins to take effect and provide a substantial coherent gain. The two figures display different mechanisms and their behaviour in the V2V urban environment. In essence, it can be inferred that Golay+SM offers a low-complexity, phase-robust solution that reduces the required SNR for a given BER, whereas 4 × 4 MIMO+MMSE provides higher throughput and better high-SNR BER performance at the cost of more RF chains and tighter CSI requirements.

**Fig 10:**
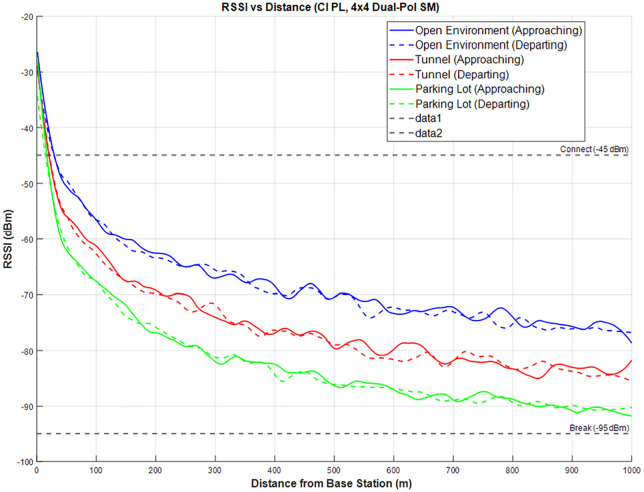
RSSI vs Distance measurement in CI Path Loss for the proposed DPSM-4×4 MIMO.

Fig 10 illustrates how RSSI changes with increasing distance in three environments: Open Environment, Tunnel, and Parking Lot, and in different directions of approaching and departing motion. The observed curves agree with the expected exponential decline in received signal strength with increasing distance from the base station, and they exhibit environment-dependent fading.

RSSI reaches a peak of approximately −30 dBm near the base station and a minimum at 500 m, indicating minimal obstruction and significant multipath propagation. In the case of the Tunnel, moderate attenuation is observed, with RSSI generally 5-8dB lower than in the open space, which is explained by the limited propagation conditions and wall reflections. The Parking Lot has the lowest RSSI of −85 dBm or lower at a 400m distance, due to a very high degree of scattering and shadowing from vehicles and surrounding structures.

There are two threshold levels: −45 dBm, which indicates the connection threshold, and −95 dBm, which indicates the link-break threshold. The RSSI at Open Environment exceeds the break threshold to approximately 900m, while in the Tunnel and Parking Lot scenarios, the approach to the break threshold occurs at shorter ranges. The relative comparison of incoming and outgoing curves reveals slight variations, which can be attributed to movement-induced fading and directional shadowing. Taken together, the graph shows that environmental complexity significantly affects link reliability and demonstrates that RSSI-based coverage performance depends heavily on context.

**Fig 11:**
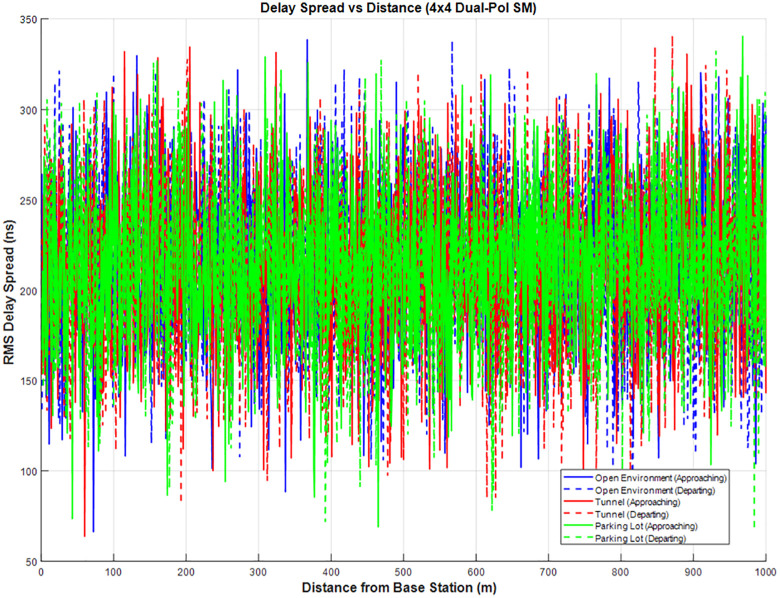
Delay Spread vs Distance measurement in CI Path Loss for the proposed DPSM-4×4 MIMO.

Fig 11 displays the RMS Delay Spread versus distance for the same three environments and directional movement, using a 4 × 4 Dual-Polarized SM setup. Delay spread is a measure of the temporal spread caused by multipath propagation and is a crucial indicator of channel frequency selectivity. The reported values range from 100 ns to 300 ns, with wide variation across locations and conditions.

The delay spread in the Tunnel scenario is moderate and is largely influenced by long reflected paths along the tunnel walls. The lowest delay spread is typically found in the Open Environment, due to the scarcity of obstructions; however, variations may occur due to reflections from distant objects. The superimposed curves indicate that the delay spread is very dynamic and responsive to micro-scale geometric variations, especially in dual-polarity SM systems. The general point of the graph is that different environments have radically different multipath richness, which implies a direct effect on system-design issues such as equalizer complexity, guard interval requirements, and modulation robustness.

Table 1 Characterizes the energy-efficiency behaviour of traditional full-multiple-input multiple-output (Full-MIMO) systems and the single-RF-chain DPSM scheme’s performance in terms of transmit SNR at a constant spectral efficiency of 8 bits per channel use (bpcu). The comparison is performed at a fixed spectral efficiency of 8 bpcu. Full-MIMO uses four QPSK streams, whereas the proposed SM scheme uses one active RF chain, 3 index bits from 8 logical ports, and 5 symbol bits from 32-QAM. The 8 bpcu comparison is made on equal spectral-efficiency footing by ensuring the baseline model uses four QPSK streams (4×2=8 bpcu), while the proposed DPSM uses 3 spatial index bits from 8 logical ports plus 5 symbol bits from 32-QAM (3+5=8 bpcu). Evidently, the DPSM scheme consistently achieves considerably higher efficiency across the entire SNR spectrum. At a reduced SNR, the improvement provided by DPSM is approximately three times that of Full-MIMO, due to reduced Power consumption in the RF chain and circuitry. With a further increase in SNR, both schemes show gradual improvements in energy efficiency; however, Full-MIMO quickly levels off as the power overhead of multiple configurations of independently operating RF chains accumulates. On the other hand, DPSM offers a much higher energy efficiency, hence highlighting the effectiveness in single-RF-chain functionality in synergy with spatial and polarization domain information mapping. Together, the table supports the evidence that the DPSM paradigm is an exemplary energy-efficient system for vehicle-to-vehicle (V2V) communications, especially in power- and hardware-constrained vehicles.

**Table 1:** Energy Efficiency Comparison at 8 bpcu, Parameters: $P_{fixed}$ = 0.50 W, $P_{RF}$ = 1.00 W.

Tx SNR (dB)	Energy Efficiency – Full-MIMO (×10⁶ bits/J)	Energy Efficiency – Proposed DPSM (×10⁶ bits/J)
**0**	**1.10**	**3.00**
**5**	**1.28**	**3.60**
**10**	**1.52**	**4.30**
**15**	**1.70**	**4.55**
**20**	**1.76**	**4.58**
**25**	**1.77**	**4.59**
**30**	**1.78**	**4.57**

**Fig 12:**
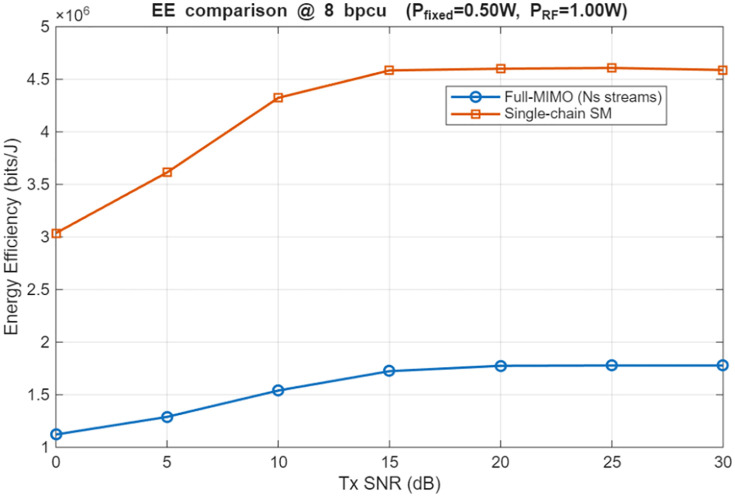
Energy Efficiency comparison for the proposed DPSM and 4×4 MIMO systems.

Fig 12 presents a comparative study of energy efficiency (EE) in bits/joule as a function of the transmit SNR for two transmission schemes. The comparison is performed with a fixed spectral efficiency of 8 bits per channel use, a fixed circuit power of P fixed = 0.50 W, and an RF-chain power of P RF = 1.00 W. Traditional Full-MIMO transmission utilises N parallel data streams, whereas the DPSM scheme describes a scenario where only one RF chain is active, but more bits conveying the information are transmitted with the aid of polarization. This is shown to result in much higher EE across the full SNR spectrum from 0 dB to 30 dB with the proposed DPSM scheme. Both schemes show EE increasing with SNR, indicating more efficient power use; however, the Full MIMO scheme quickly levels off at high SNR due to the cumulative circuit and RF-chain power demands of multiple active streams. In its turn, the specified DPSM scheme achieves a far greater EE plateau, which underlines the advantage of a single-RF-chain activation alongside the mapping of polarization domains of information. Combined, the figure substantiates that the presented DPSM scheme provides a highly energy-efficient alternative to Full-MIMO systems, making it particularly attractive for green communications and wireless systems that struggle with power consumption.

**Fig 13:**
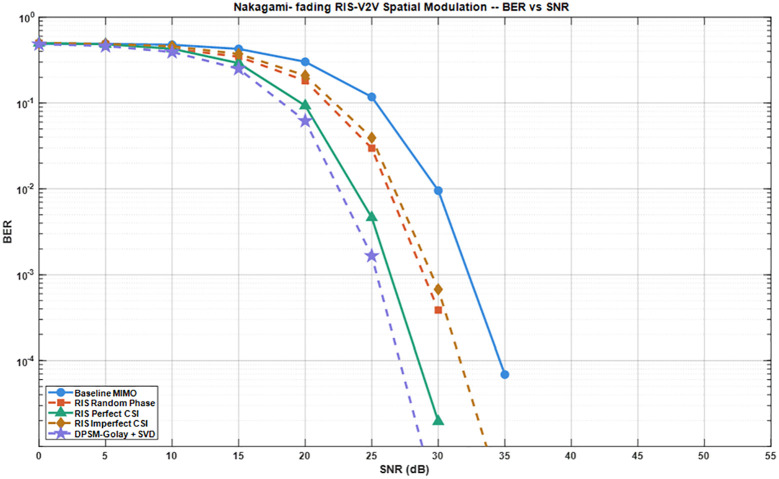
DPSM and 4×4 MIMO systems in a realistic slow fading Nakagami fading environment.

Fig 13 shows BER versus SNR for the five schemes under Nakagami-*m* fading in the RIS-V2V spatial modulation scenario; the baseline MIMO curve (blue) falls from BER ≈ 1.2 × 10 ⁻ ¹ at 25 dB to ≈ 1.0 × 10 ^⁻^ ² at 30 dB and ≈ 1.0 × 10 ⁻ ⁴ at 35 dB, an accelerating, steepening slope characteristic of increasing diversity order at higher SNR rather than an inconsistency. Anchoring all comparative claims to single, labelled operating points: at SNR = 25 dB the proposed DPSM-Golay + SVD scheme (purple) achieves BER ≈ 1.5 × 10 ^⁻^ ³ against baseline MIMO’s ≈ 1.2 × 10 ⁻ ¹, an **80 × BER reduction**; at the BER = 10 ^⁻^ ³ threshold, DPSM-Golay + SVD reaches this level at ≈ 25 dB versus baseline MIMO’s interpolated ≈ 32 dB, a **7 dB SNR gain**. The RIS Random Phase and RIS Imperfect CSI curves are categorically distinct: Random Phase assigns each RIS element an i.i.d. phase from *U*[0, 2π] with receiver channel knowledge, the Imperfect CSI uses an optimized MRC precoder with a known, tracked phase reference but a noisy channel amplitude/direction estimate; the two curves converge in the 20–27 dB range. Because estimation error dominates at moderate SNR, then separate above ≈27 dB as pilot accuracy improves, with both eventually outperformed by RIS Perfect CSI and DPSM-Golay + SVD.

## 5. Conclusions

This work developed a low-energy consumption RIS-aided dual-polarized spatial modulation (RIS-DPSM) framework for vehicle-to-vehicle (V2V) communication. This architecture enables single-active-RF-chain transmission in the proposed link-level SM model, while the separate 8 bpcu benchmark shows the corresponding energy-efficiency gain over a 4-stream QPSK Full-MIMO baseline. Compared with conventional full-MIMO systems, the proposed framework leverages spatial and polarization indexing, along with RIS reconfigurability, to enable single-RF-chain transmission and reduce the complexity of the RF front end, circuit power consumption, and inter-RF-chain synchronization. The proposed DPSM framework achieves the energy efficiency of $\left(\text{3}.\text{0}\text{0}\times \;{\text{1}\text{0}}^{\text{6}}\right)$ bits/J at 0 dB, when compared to $\left(\text{1}.\text{1}\text{0}\times \;{\text{1}\text{0}}^{\text{6}}\right)$ bits/J, of the full-MIMO benchmark. The RIS-assisted links outperform the non-RIS system in RSSI, RSRP, and RSRQ, and achieve substantial gains in rain attenuation and keyhole channel performance.

An important feature of the proposed framework is the RIS phase-optimization strategy. This combines phase alignment via singular-value decomposition with Golay-complementary-sequence-aided reflection control. This allows for near-coherent combining of the received polarized components while maintaining the orthogonality of the polarization domains. Thus, polarization diversity is enhanced and the overall end-to-end channel gain is improved.

The results verify that RIS-assisted DPSM has better energy efficiency compared to traditional full-MIMO solutions in the medium-to-high SNR range. These benefits are mainly attributed to the single-RF-chain functionality, design of reduced circuit/RF power consumption, and efficient polarization-domain multiplexing. The proposed framework has the potential to support dense urban vehicular networks, in which RIS panels can be integrated into the roadside infrastructure and coordinated by a single MEC node, shifting most of the control and computation from vehicles to the roadside. Thus, RIS-DPSM is a desirable solution for low complexity, low energy consumption and high reliability for V2V communication.
